# Pharmacokinetics, distribution, metabolism, and excretion of body-protective compound 157, a potential drug for treating various wounds, in rats and dogs

**DOI:** 10.3389/fphar.2022.1026182

**Published:** 2022-12-14

**Authors:** Lei He, Donglin Feng, Hui Guo, Yueyuan Zhou, Zhaozhao Li, Kuo Zhang, Wangqian Zhang, Shuning Wang, Zhaowei Wang, Qiang Hao, Cun Zhang, Yuan Gao, Jintao Gu, Yingqi Zhang, Weina Li, Meng Li

**Affiliations:** ^1^ State Key Laboratory of Cancer Biology, Department of Biopharmaceutics, School of Pharmacy, Air Force Medical University, Xi’an, China; ^2^ School of Pharmacy, Shaanxi University of Chinese Medicine, Xi’an, China

**Keywords:** BPC157, pharmacokinetics, absorption, distribution, metabolism, excretion, wounds

## Abstract

Body-protective compound (BPC) 157 demonstrates protective effects against damage to various organs and tissues. For future clinical applications, we had previously established a solid-phase synthesis process for BPC157, verified its biological activity in different wound models, and completed preclinical safety evaluations. This study aimed to investigate the pharmacokinetics, excretion, metabolism, and distribution profiles of BPC157. After a single intravenous (IV) administration, single intramuscular (IM) administrations at three doses in successive increments along with repeated IM administrations, the elimination half-life (t_1/2_) of prototype BPC157 was less than 30 min, and BPC157 showed linear pharmacokinetic characteristics in rats and beagle dogs at all doses. The mean absolute bioavailability of BPC157 following IM injection was approximately 14%–19% in rats and 45%–51% in beagle dogs. Using [^3^H]-labeled BPC157 and radioactivity examination, we proved that the main excretory pathways of BPC157 involved urine and bile. [^3^H]BPC157 was rapidly metabolized into a variety of small peptide fragments *in vivo*, thus forming single amino acids that entered normal amino acid metabolism and excretion pathways. In conclusion, this study provides the first analysis of the pharmacokinetics of BPC157, which will be helpful for its translation in the clinic.

## 1 Introduction

Body-protective compound (BPC) 157 is a peptide isolated from human gastric juice (Sikiric et al., 1993). BPC157 comprises 15 amino acids (Gly-Glu-Pro-Pro-Pro-Pro-Gly-Lys-Pro-Ala-Asp-Asp-Ala-Gly-Leu-Val) and has a molecular weight of 1419 Da. Also known as BPC-15, PL-10, PLD-116, or PL14736 (Keremi et al., 2009), BPC157 has demonstrated remarkable potential as a therapeutic agent for severe trauma and stress damage and can promote the healing of wounds, ligament injuries, tendon injuries, and fractures. BPC157 exerts a significant protective effect on various tissues and organs, such as the esophagus, stomach, duodenum (Drmic et al., 2017), colorectal mucosa (Duzel et al., 2017), liver, pancreas (Konturek and Brzozowski, 2008), muscle (Lai et al., 2019), cornea (Lazic et al., 2005), heart (Sikiric et al., 2016) and nerves (Grabarevic et al., 1997; Klicek et al., 2013; Wang et al., 2019). Apart from its protective effect against multiple organ injuries, BPC157 has also demonstrated cytoprotective (Sikiric et al., 2018) and anti-inflammatory properties and plays a role in maintaining epithelial integrity (Mota et al., 2018). Although the mechanism of action of BPC157 remains unclear, BPC157 has demonstrated significant effects at very low doses with very good stability (Sikiric et al., 2018). It can be stored at room temperature and is resistant to hydrolysis, enzyme digestion, and even gastric juice. Based on the stability and pleiotropy of BPC157, it is an ideal candidate for the treatment of all types of severe trauma and may be superior to the widely used cytokine drugs in wound therapy.

Previously, we established a solid-phase synthesis process for BPC157 (Xue et al., 2004) and verified its biological activity in rat gastric ulcer models and various skin wound models (Huang et al., 2015). At the same time, our preclinical safety evaluation studies showed that BPC157 was well tolerated and did not demonstrate any serious toxic effects in mice, rats, rabbits, or dogs (Xu et al., 2020). For evaluating its future clinical use as a therapeutic drug and follow-up clinical trials, the present study was undertaken to evaluate the pharmacokinetics, tissue distribution, metabolism, and drug excretion of BPC157 in Sprague-Dawley (SD) rats and beagle dogs as well as in associated *in vitro* studies. The experiments were performed according to the criteria of the new investigational drug application. This study is the first preclinical pharmacokinetic study of BPC157, and the results are of considerable importance, as they demonstrate not only the pharmacokinetic parameters of BPC157 but also show the implications for its systemic application as a novel drug for various injuries.

## 2 Results

### 2.1 Pharmacokinetic studies of BPC157 in rats

The effective dose of BPC157 for the treatment of various injuries in mice, rats, and rabbits ranges from 6 to 50 μg/kg (Huang et al., 2015; Mota et al., 2018; Sikiric et al., 2018). Our proposed clinical dose of BPC157 was 200 µg/person/day, and its equivalent dose in rats was 20 μg/kg (converted based on body surface area). Therefore, we performed pharmacokinetic studies of BPC157 in rats following a single intravenous (IV) administration of 20 μg/kg, single intramuscular (IM) administration of doses 20, 100, or 500 μg/kg, and repeated IM administrations of 100 μg/kg of BPC157 for seven consecutive days. The administration of BPC157 was well tolerated by all rats, and no visual signs of toxicity were observed, consistent with our previous safety evaluation studies (Xu et al., 2020). In addition, no noticeable difference in the plasma concentration of BPC157 was observed between male and female rats.

**FIGURE 1:**
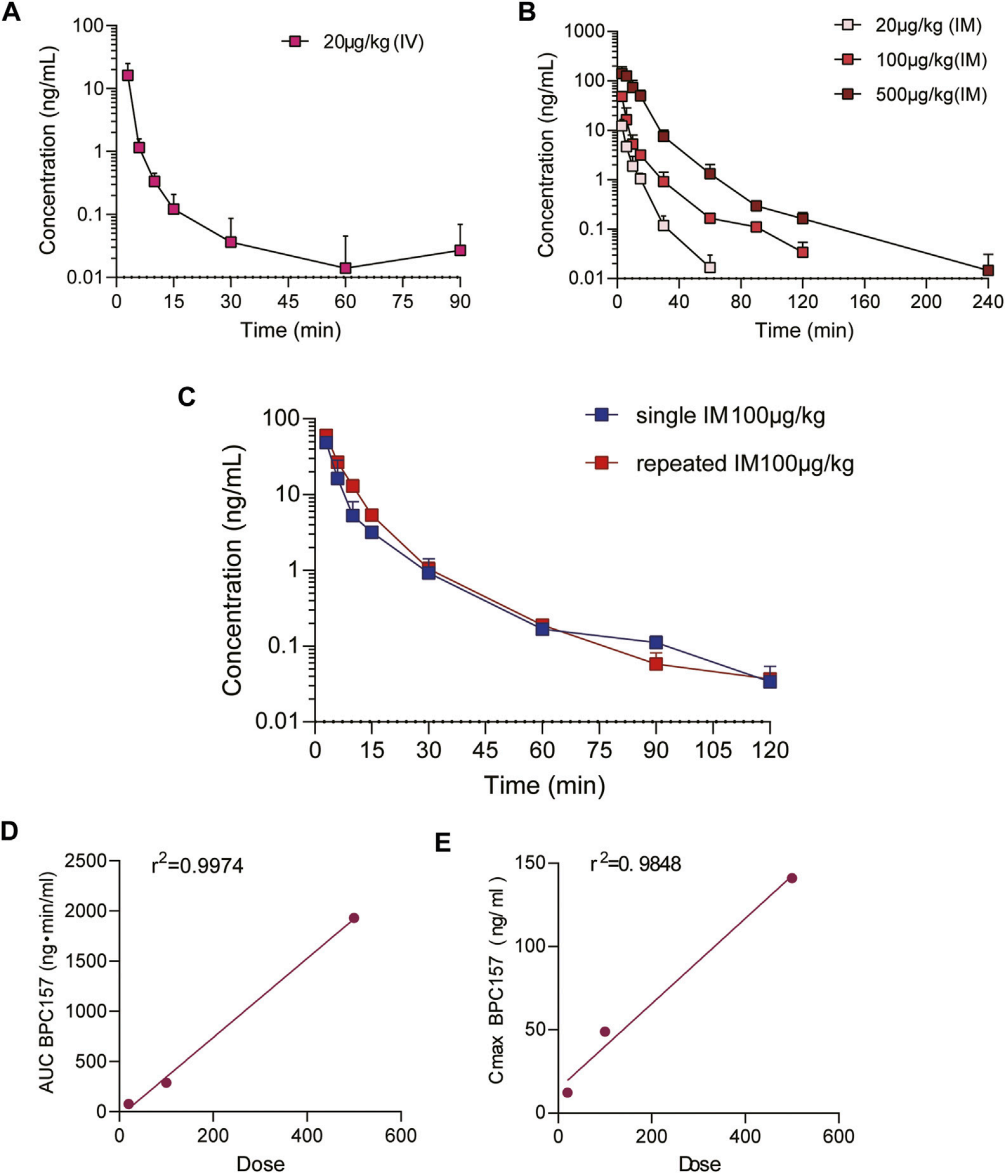
Pharmacokinetic studies of BPC157 in rats (mean ± SD, *n* = 6). **(A)** Total plasma concentration versus time profiles of BPC157 following intravenous administration at a dose of 20 μg/kg; **(B)** Total plasma concentration versus time profiles of BPC157 following intramuscular administration at the doses of 20, 100, or 500 μg/kg; **(C)** Total plasma concentration versus time profiles of BPC157 following repeat intramuscular administration at a dose of 100 μg/kg for seven consecutive days (red) or a single intramuscular administration at a dose of 100 μg/kg (blue). **(D)** Linear regression between BPC157 AUC and doses evaluated following intramuscular administration of doses 20, 100, or 500 μg/kg. **(E)** Linear regression between BPC157 C_max_ and doses evaluated following intramuscular administration of doses 20, 100, or 500 μg/kg. The goodness of fit was estimated by the coefficient of determination (r^2^). Data are representative of three independent experiments.

The mean (+SD) plasma concentration of BPC157 versus time curves following administration of various BPC157 doses in rats are shown in Figures 1A–C, and the corresponding pharmacokinetic parameters are presented in Tables 1–Tables 3. After a single IV administration, BPC157 was rapidly eliminated from the plasma of rats, and the average elimination half-life (t_1/2_) was 15.2 min. The average area under the plasma concentration-time curve (AUC_0–t_) was 399 ng min/ml. After single IM administrations of doses 20, 100, or 500 μg/kg, the peak time (T_max_) of each dose was 3 min. The maximum concentrations (C_max_) of each dose were 12.3, 48.9, and 141 ng/ml, respectively, and the AUC_0–t_ values were 75.1, 289, and 1930 ng min/ml, respectively. Linear relationships were observed between AUC_0–t_ and BPC157 doses, as well as between C_max_ and BPC157 doses (Figures 1D,E). The absolute bioavailability after IM administration of each dose was 18.82%, 14.49%, and 19.35%, respectively. After repeated IM administration of BPC157 at 100 μg/kg for seven consecutive days, the plasma concentration versus time curve (Figure 1C) and pharmacokinetic parameters (Table 3) were similar to those observed after a single IM injection at a dose of 100 μg/kg, except for a slight increase in C_max_ and AUC_0–t_. The aforementioned results showed that BPC157 reached its peak rapidly in rats and was rapidly eliminated after reaching its peak. The prototype drug could not be detected 4 h after administration, and its elimination half-life was less than 30 min. BPC157 showed linear pharmacokinetic characteristics in rats at the experimental dose.

**TABLE 1:** Pharmacokinetic parameters of BPC157 in rats following a single intravenous administration (mean ± SD, *n* = 6).

AUC_0–90min_	AUC_0-∞_	MRT	t_1/2_	V_ss_	CL
(ng∙min/ml)	(ng∙min/ml)	(min)	(min)	(ml/kg)	(ml/min/kg)
399	400	0.727	15.2	36.4	50.1

AUC_0–90min_, area under the concentration/time curve at last observation; AUC_0–∞_, area under the concentration/time curve from 0 h to infinity; t_1/2_, half-life; V_ss_, volume of distribution (steady state); CL, clearance.

**TABLE 2:** Pharmacokinetic parameters of BPC157 in rats following intramuscular administrations of doses ranging between 5 and 120 μg/kg (mean ± SD, *n* = 6).

Dosage	T_max_	C_max_	AUC_0–t_	AUC_0–∞_	MRT	t_1/2_	F_a_
(μg/kg)	(min)	(ng/ml)	(ng·min/ml)	(ng·min/ml)	(min)	(min)	(%)
20	3.00	12.3	75.1	75.3	7.27	7.87	18.82
100	3.00	48.9	289	290	9.61	17.1	14.49
500	3.00	141	1930	1931	12.8	29.7	19.35

T_max_, time to peak concentration of drug in plasma; C_max_, peak concentration of drug in plasma; F_a_ = (AUC_0–t_IM∙DoseIV)/(AUC_0–t_IV∙DoseIM) × 100%.

**TABLE 3:** Pharmacokinetic parameters of BPC157 in rats following repeat intramuscular administration of 100 μg/kg BPC157 for seven consecutive days (mean ± SD, *n* = 6).

T_max_	C_max_	AUC_0–t_	AUC_0–∞_	t_1/2_	C_ssav_	C_ssmin_
(min)	(ng/ml)	(ng·min/ml)	(ng·min/ml)	(min)	(ng/ml)	(ng/ml)
3.00	60.4	418	419	18.5	0.290	0

C_ssav_ = AUC_0–t_/τ, τ is the interval of administration;C_ssmin_=(C_-48h_ + C_-24h_ + C_0h_)/3.

### 2.2 Pharmacokinetic studies of BPC157 in beagle dogs

Our proposed clinical dose of BPC157 was 200 µg/person/day, and its equivalent dose in dogs was 6 μg/kg (converted based on body surface area). Therefore, we performed pharmacokinetic studies of BPC157 in beagle dogs following single IV administration at a dose of 6 μg/kg, single IM administration at doses of 6, 30, or 150 μg/kg, and repeated IM administration at a dose of 30 μg/kg for seven consecutive days. The administration of BPC157 was well tolerated by all dogs, and no visual signs of toxicity were observed, which was consistent with our previous safety evaluation studies. No noticeable difference in the plasma concentration of BPC157 was found between male and female dogs.

**FIGURE 2:**
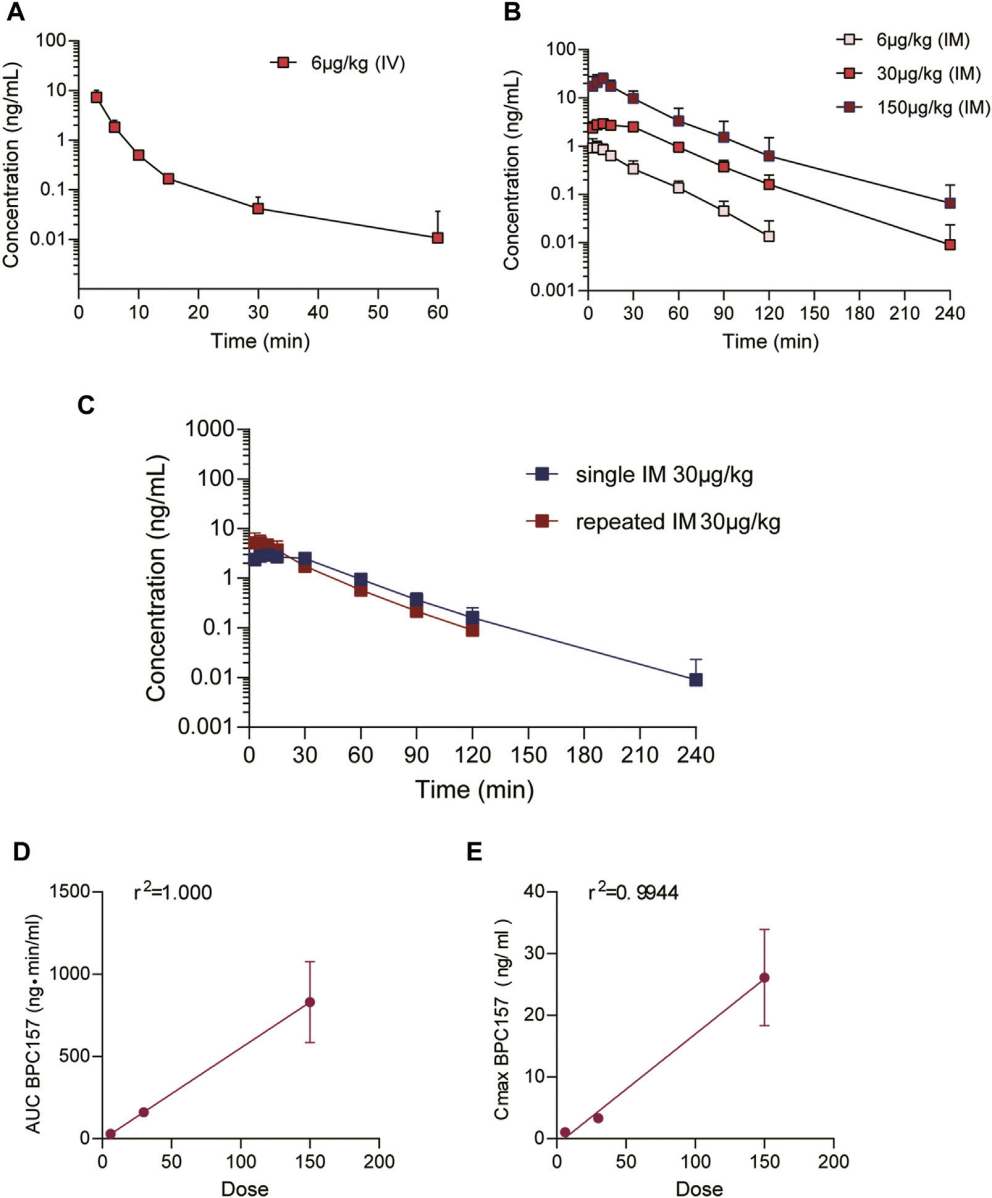
Pharmacokinetic studies of BPC157 in beagle dogs (mean ± SD, *n* = 6). **(A)** Total plasma concentration versus time profiles of BPC157 following intravenous administration at a dose of 6 μg/kg; **(B)** Total plasma concentration versus time profiles of BPC157 following intramuscular administration of doses 6, 30, or 150 μg/kg; **(C)** Total plasma concentration versus time profiles of BPC157 following repeated intramuscular administration at a dose of 30 μg/kg for seven consecutive days (red) or a single intramuscular administration at a dose of 30 μg/kg (blue). **(D)** Linear regression analysis between BPC157 AUC and doses following intramuscular administration of doses 6, 30, or 150 μg/kg. **(E)** Linear regression analysis between BPC157 C_max_ and doses following intramuscular administration of doses 6, 30, or 150 μg/kg. The goodness of fit was estimated by coefficients of determination (r^2^). Data are representative of three independent experiments.

The mean (+SD) BPC157 plasma concentration versus time curves following administration of various BPC157 doses in dogs are shown in Figures 2A–C, and the corresponding pharmacokinetic parameters are presented in Tables 4–Tables 6. After single IV administration, the t_1/2_ and AUC_0–t_ of BPC157 in dogs were 5.27 min and 76.4 ± 30.2 ng min/ml. After single IM administration at doses of 6, 30, or 150 μg/kg, the T_max_ values of each dose were 6.33, 8.67, and 8.17 min, respectively. The C_max_ values of each dose were 1.05 ± 0.429, 3.30 ± 0.508, and 26.1 ± 7.82 ng/ml, respectively, and the AUC_0–t_ values were 29.0 ± 2.68, 160 ± 21.0, and 830 ± 247 ng min/mL respectively. Linear relationships were observed between AUC_0–t_ and BPC157 doses, as well as between C_max_ and BPC157 doses (Figures 2D,E). The absolute bioavailability observed after IM administration of each dose in dogs was 45.27%, 47.64%, and 50.56%, respectively. After repeated IM administration of BPC157 at 30 μg/kg for seven consecutive days, the plasma concentration versus time curve was similar to that observed after a single IM injection of 30 μg/kg (Figure 2C). However, the pharmacokinetic parameters after repeated IM administration changed slightly compared to those observed after a single IM injection, with a small decrease in C_max_ and t_1/2_ and an increase in T_max_. The area under the curve (AUC) values did not change significantly (Table 6). The aforementioned results showed that BPC157 reached its peak rapidly in beagle dogs and was rapidly eliminated after reaching its peak. The prototype drug could not be detected 4 h after administration, and its elimination half-life was less than 30 min. BPC157 showed linear pharmacokinetic characteristics in beagle dogs at the experimental dose.

**TABLE 4:** Pharmacokinetic parameters of BPC157 in dogs following a single intravenous administration (mean ± SD, *n* = 6).

AUC_0–tmin_	AUC_0-∞_	MRT	t_1/2_	V_ss_	CL
(ng∙min/ml)	(ng∙min/ml)	(min)	(min)	(ml/kg)	(ml/min/kg)
76.4 ± 30.2	76.9 ± 30.2	2.49 ± 0.822	5.27 ± 2.25	243 ± 162	90.8 ± 40.1

AUC_0–90min_, area under the concentration/time curve at last observation; AUC_0–∞_, area under the concentration/time curve from 0 h to infinity; t_1/2_, half-life; V_ss_, volume of distribution (steady state); CL, clearance.

**TABLE 5:** Pharmacokinetic parameters of BPC157 in dogs following intramuscular administration of doses ranging between 6 and 150 μg/kg (mean ± SD, *n* = 6).

Dosage (μg/kg)	T_max_	C_max_	AUC_0-t_	AUC_0-∞_	MRT	t_1/2_	F_a_
	(min)	(ng/ml)	(ng·min/ml)	(ng·min/ml)	(min)	(min)	(%)
6	6.33 ± 3.14	1.05 ± 0.429	29.0 ± 2.68	30.0 ± 3.11	25.8 ± 7.35	20.0 ± 5.53	45.27 ± 24.85
30	8.67 ± 5.54	3.30 ± 0.508	160 ± 21.0	164 ± 21.5	37.0 ± 7.77	25.5 ± 7.08	47.64 ± 18.09
150	8.17 ± 2.99	26.1 ± 7.82	830 ± 247	831 ± 246	31.4 ± 15.2	29.3 ± 5.06	50.56 ± 27.01

T_max_, time to peak concentration of drug in plasma; C_max_, peak concentration of drug in plasma; F_a_ = (AUC_0–t_IM∙DoseIV)/(AUC_0–t_IV∙DoseIM) × 100%.

**TABLE 6:** Pharmacokinetic parameters of BPC157 in dogs following repeated intramuscular administration at a dose of 30 μg/kg for seven consecutive days (mean ± SD, *n* = 6).

T_max_ (min)	C_max_ (ng/ml)	AUC_0–t_ (ng·min/ml)	AUC_0-∞_ (ng·min/ml)	t_1/2_ (min)	C_ssav_ (ng/ml)	C_ssmin_ (ng/ml)
10.5 ± 10.5	5.89 ± 2.41	155 ± 25.2	158 ± 26.0	19.6 ± 3.72	0.108 ± 0.0177	0.00285 ± 0.00698

C_ssav_ = AUC_0–t_/τ, τ is the interval of administration; C_ssmin_ = (C_-48h_ + C_-24h_ + C_0h_)/3.

### 2.3 Excretion, metabolism, and tissue distribution of BPC157

**FIGURE 3:**
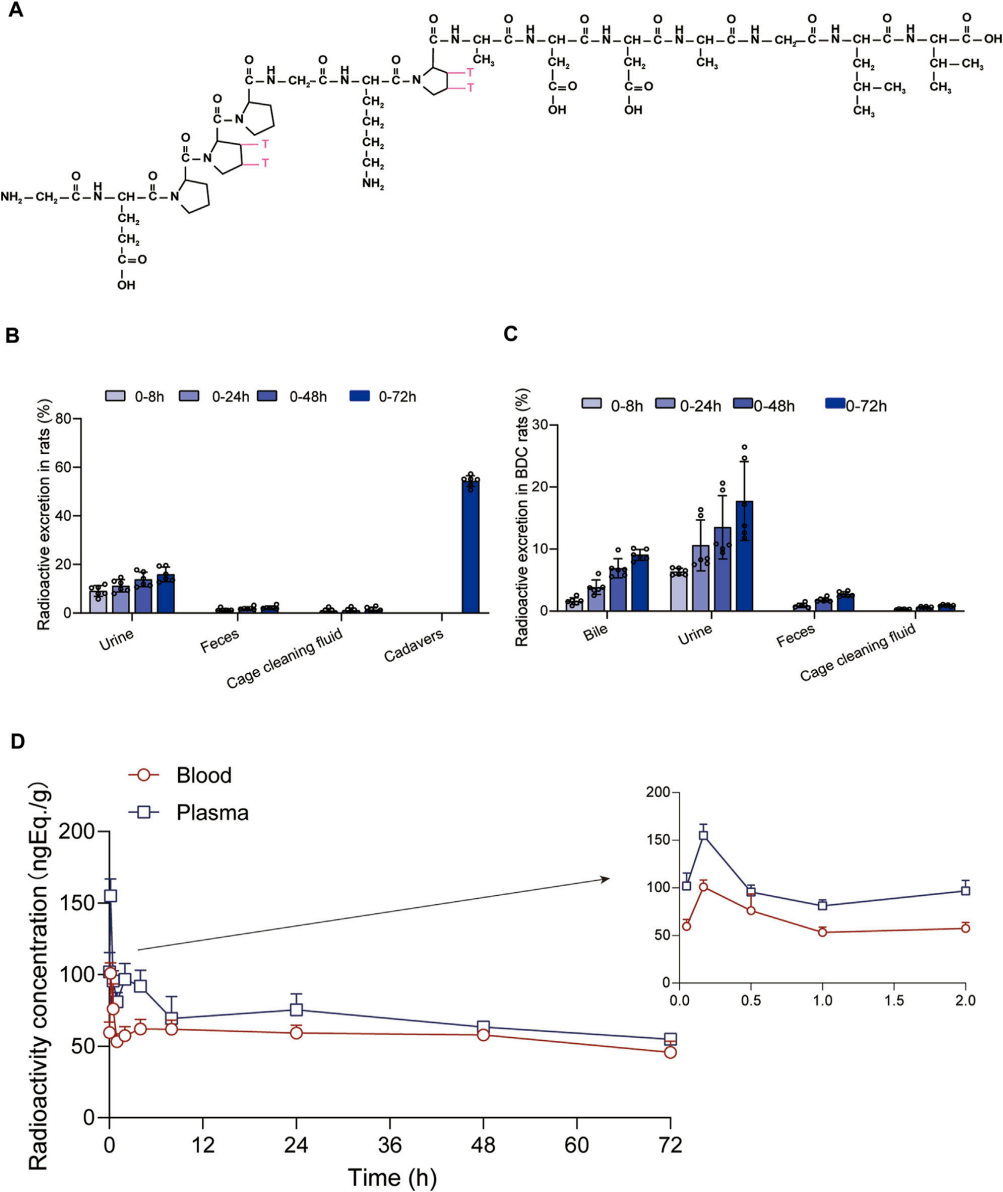
Evaluation of BPC157 excretion in rats by examination of total radioactivity. **(A)** The structure of [^3^H]-labeled BPC157. [^3^H]-labeled sites are indicated by T (red). **(B)** The average recovery of total radioactivity in the urine, feces, cage cleaning fluid, and cadavers during 0–72 h after [^3^H]BPC157 administration in intact rats (mean ± SD, *n* = 6). **(C)** The average recovery of total radioactivity in the urine, feces, and cage cleaning fluid collected during 0–72 h after [^3^H]BPC157 administration in bile duct cannulated rats (mean ± SD, *n* = 6). **(D)** Total radioactivity concentration versus time curve for [^3^H]BPC157 in jugular vein cannulated rats following a single intramuscular injection at a dose of 100 µg/300 μCi/kg (mean ± SD, *n* = 6). Data are representative of three independent experiments.

In the aforementioned studies, we characterized the pharmacokinetic profile of prototype BPC157 using high-performance liquid chromatography (HPLC) in rats and dogs. Next, we evaluated the excretion, metabolism, and tissue distribution of BPC157 in rats after a single IM injection of 100 µg/300 μCi/kg [^3^H]BPC157. [^3^H]BPC157 was well tolerated by all rats, and no visual signs of toxicity were observed. Prolines of BPC157 were labeled with [^3^H] and the structure of [^3^H]-labeled BPC157 is shown in Figure 3A.

#### 2.3.1 BPC157 excretion in rats evaluated by examination of total radioactivity

The average recovery rates of total radioactivity in urine, feces, and cage cleaning fluid collected from 0 to 72 h after [^3^H]BPC157 administration in intact rats were 15.88% ± 2.99%, 2.25% ± 0.67%, and 1.41% ± 1.04%, respectively, and the proportion of residual radioactivity in the cadavers was 54.31% ± 3.04% (Table 7; Figure 3B). Furthermore, in bile duct‒cannulated (BDC) rats, the average recovery rates of total radioactivity in bile, urine, feces, and cage cleaning fluid collected during 72 h after dosing were 9.08% ± 0.86%, 17.77% ± 6.35%, 2.73% ± 0.40%, and 0.91% ± 0.13%, respectively (Table 8; Figure 3C). These results suggest that urinary excretion is the dominant route of elimination following IM administration of BPC157.

**TABLE 7:** The recovery of total radioactivity in the urine, feces, and cage cleaning fluid during 0–72 h after intramuscular administration of [3H]BPC157 in rats.

Time interval (hour)	Recovery of radioactivity (%)			
	Urine	Feces	Cage cleaning fluid	Cadavers
0–8	9.11 ± 2.21	—	—	—
0–24	11.25 ± 2.54	1.31 ± 0.54	0.94 ± 0.91	—
0–48	13.84 ± 3.00	1.92 ± 0.61	1.08 ± 0.93	—
0–72	15.88 ± 2.99	2.25 ± 0.67	1.41 ± 1.04	54.31 ± 2.27

**TABLE 8:** The recovery of total radioactivity in bile, urine, feces, and cage cleaning fluid during 0–72 h after intramuscular administration of [3H]BPC157 in BDC rats.

Time interval (hour)	Recovery of radioactivity (%)			
	Bile	Urine	Feces	Cage cleaning fluid
0–8	1.61 ± 0.55	6.37 ± 0.54	—	—
0–24	3.86 ± 1.18	10.62 ± 4.10	0.92 ± 0.34	0.29 ± 0.08
0–48	6.92 ± 1.55	13.53 ± 5.11	1.84 ± 0.33	0.59 ± 0.17
0–72	9.08 ± 0.86	17.77 ± 6.35	2.73 ± 0.40	0.91 ± 0.13

#### 2.3.2 Plasma pharmacokinetic parameters of BPC157 in rats evaluated based on total radioactivity

After a single IM injection of 100 µg/300 μCi/kg [3H]BPC157 in jugular vein‒ cannulated (JVC) rats, the total radioactivity concentrations in the whole blood and plasma were similar between males and females. The total radioactivity concentration versus time curve is shown in Figure 3D. The corresponding pharmacokinetic parameters are listed as follows: T_max_ = 0.167 h, C_max_ = 155 ± 11.8 ng Eq./ml, AUC_0–t_ = 4945 ± 417 h ng Eq./ml, AUC_0–∞_ = 12956 ± 2074 h•ng Eq./ml, average residence time (MRT_0-t_) of plasma total radioactivity = 33.2 ± 1.03 h, and t_1/2_ = 102 h (Table 9). Compared with the results of prototype BPC157 in rats, the pharmacokinetic parameters of [^3^H]BPC157 evaluated based on total radioactivity changed markedly, indicating the significant metabolism and decomposition process of BPC157 *in vivo*.

**TABLE 9:** Plasma pharmacokinetic parameters examined based on total radioactivity following a single intramuscular administration of 100 µg/300 μCi/kg of [3H]BPC157 in rats (mean ± SD, *n* = 6).

T_max_ (h)	C_max_ (ng-Eq./ml)	AUC_0–t_ (ng-Eq./ml)	AUC_0–∞_ (h·ng·min/ml)	t_1/2_ (h)	MRT_0–t_ (h)
0.167	155 ± 11.8	4945 ± 417	12956 ± 2074	102 ± 32	33.2 ± 1.03

#### 2.3.3 Metabolite analysis of BPC157

**FIGURE 4:**
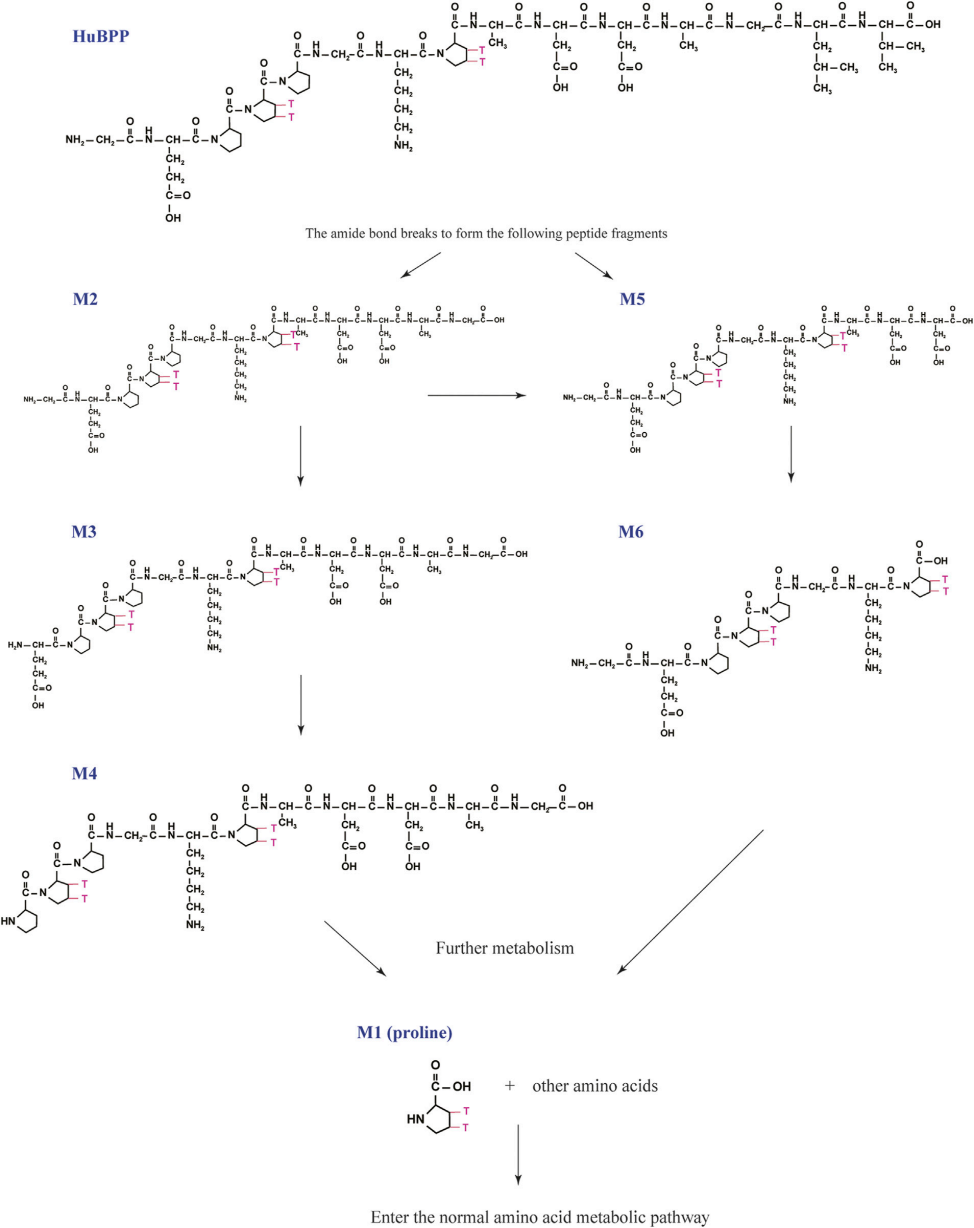
Speculated metabolic pathways of BPC157 *in vivo*. BPC157 gradually degraded into small molecular fragments and finally into single amino acids, which entered the metabolic circulation *in vivo*.

**FIGURE 5:**
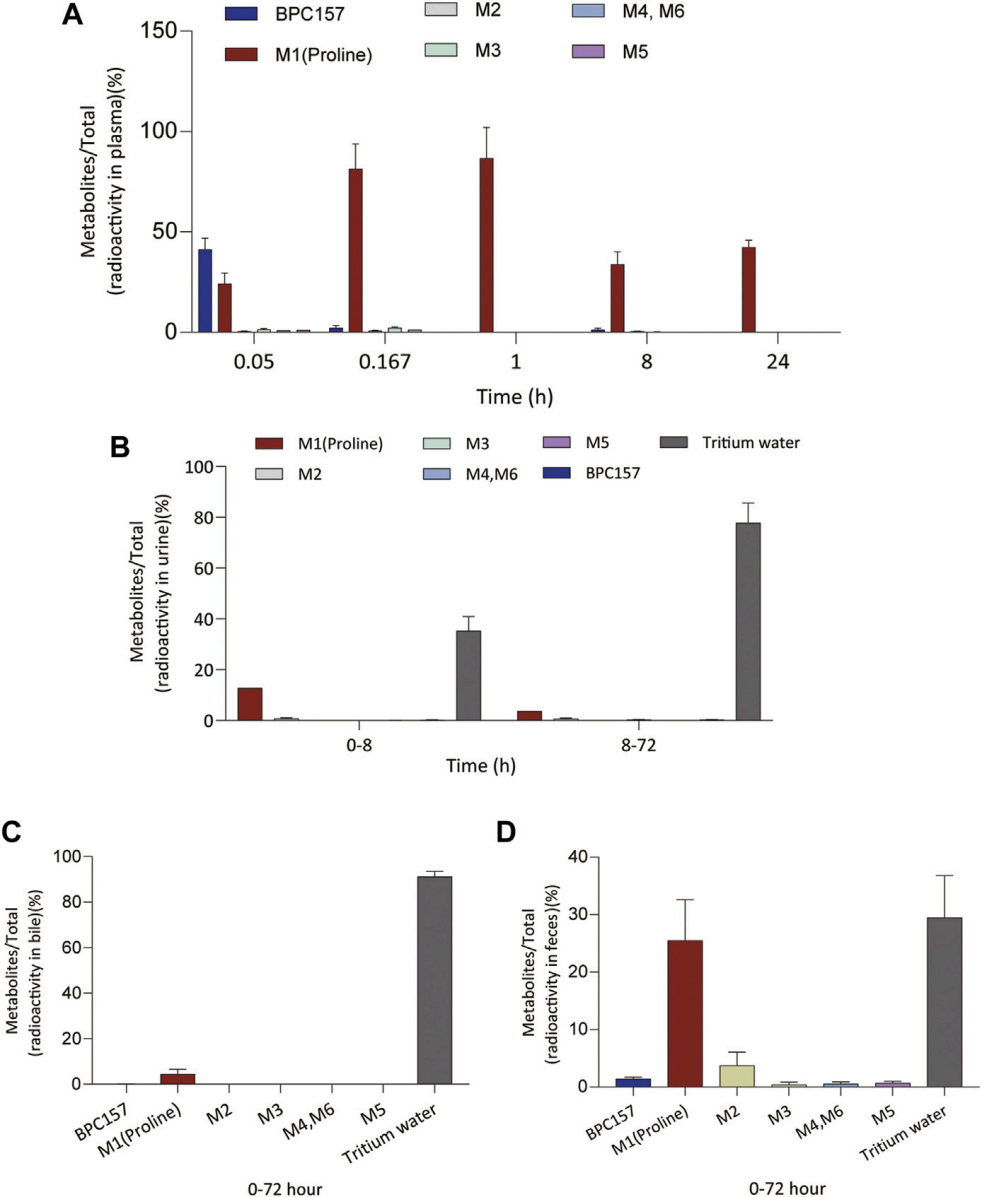
Changes in the proportion of six metabolites (M1‒M6) with time in plasma **(A)**, urine **(B)**, bile **(C)**, and feces **(D)** after a single intramuscular administration of 100 µg/300 μCi/kg of [3H]BPC157 in rats (mean ± SD, *n* = 6). Data are representative of three independent experiments.

We analyzed the metabolites of [^3^H]BPC157 in rat plasma, bile, urine, and feces using the samples collected during the aforementioned extraction and plasma pharmacokinetic studies. First, using HPLC and HPLC-associated radioactive detector, we identified six radioactive components, in addition to prototype [^3^H]BPC157, in rat plasma collected at 0.05, 0.167, 1, 8, and 24 h after administration (Supplementary Figure S1). Through high-performance liquid chromatography-tandem mass spectrometry (LC-MS/MS)-based molecular weight identification, standard molecular weight comparison, and characteristic HPLC profiles of the [^3^H]proline and [^3^H]BPC157 standards (Supplementary Figure S2), we speculated the structures of these six components and designated them as M1–M6 (Table 10). M1 was identified as [^3^H]proline and M2–M6 were identified as a variety of small molecular peptides generated upon the degradation of [3H]BPC157. Based on the structures of the M1–M6 metabolites, we proposed the metabolic process of BPC157 *in vivo* (Figure 4). The proportions of M1–M6 and [^3^H]BPC157 in plasma radioactive components at different sampling times demonstrated that [^3^H]BPC157 was the main plasma component at 0.05 h (3 min) after administration and was subsequently degraded into small molecular peptide fragments in the following 0.167 h (10 min) (Figure 5A). At 1 h after administration, [^3^H]proline accounted for 86.65% of the plasma radioactive components. Subsequently, the proportion of tritium water increased and that of [^3^H]proline decreased gradually with time.

**TABLE 10:** Structures of six metabolites identified by high-performance liquid chromatography-tandem mass spectrometry in rat plasma, bile, urine, and feces following a single intramuscular administration of 100 µg/300 μCi/kg of [3H]BPC157.

Designation	Structure	Molecular weight (Da)	Retention time (min)
BPC157	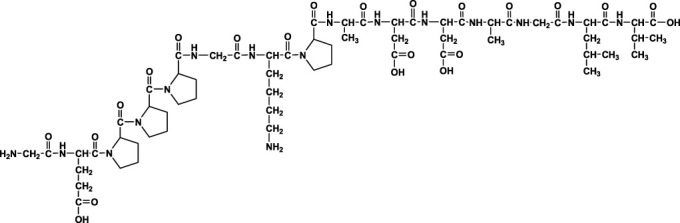	1419.5	∼39.8
M1(Proline)	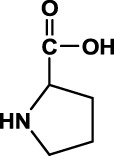	115.1	∼5.1
M2	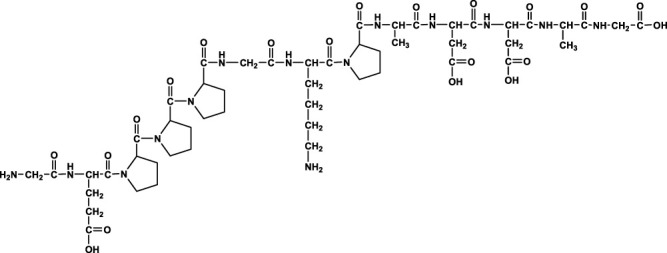	1207.3	∼19.5
M3	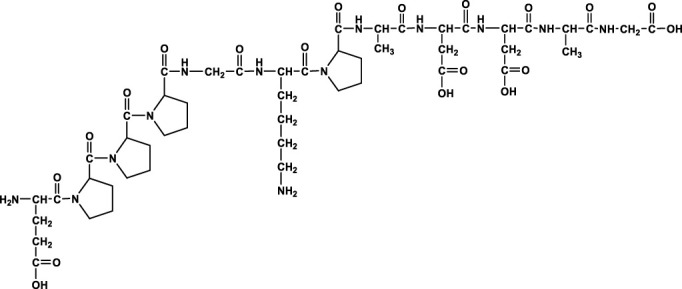	1150.2	∼19.4
M4	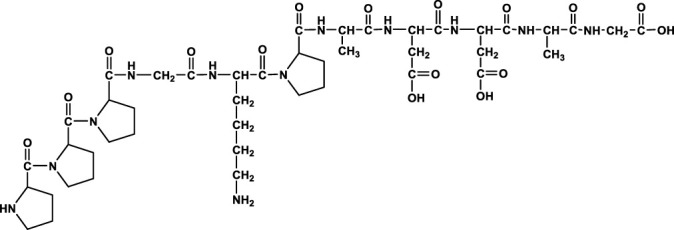	1021.1	∼19.1
M5	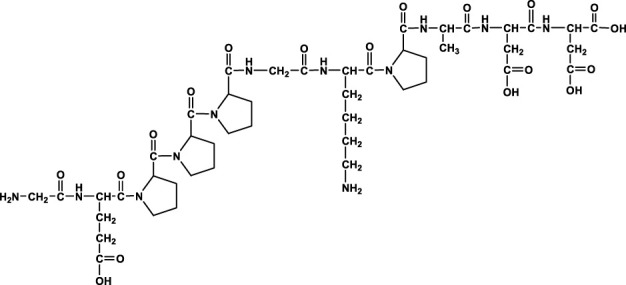	1079.1	∼18.7
M6	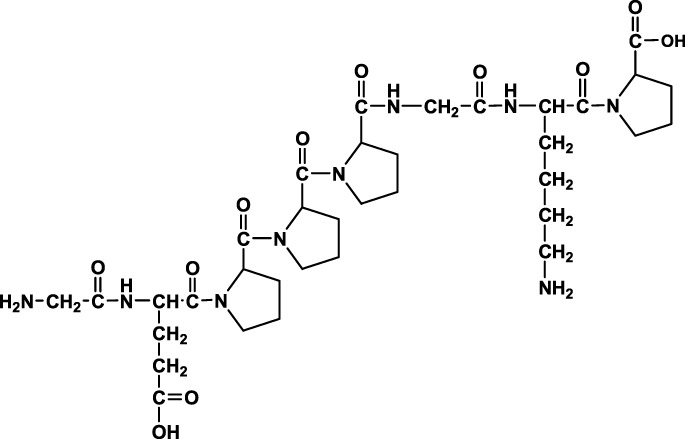	777.9	∼19.0

Rt, Retention time on LC-MS/MS.

Next, we analyzed the main metabolites of [^3^H]BPC157 in urine collected from 0 to 8 h and from 8 to 72 h and in bile and feces collected from 0 to 72 h after administration. No new metabolites were found in urine, bile, and fecal samples other than the six components found in the plasma. In the mixed urine samples collected from 0 to 8 h, the content of [^3^H]proline (M1), the main metabolite, was higher, accounting for 13.9% (female) and 11.7% (male) of the total radioactivity. In mixed urine samples collected between 8 and 72 h, the proportion of tritium water was higher, accounting for 69.5% (female) and 75.3% (male) of the total radioactivity, and [^3^H]proline (M1) accounted for 3.11% (female) and 4.17% (male) of the total radioactivity (Figure 5B). The total radioactivity excretion in mixed bile samples collected between 0 and 72 h was low, and tritium water was primarily detected, accounting for 91.2% (females) and 91.0% (males) of the sample. The main metabolite, [^3^H]proline (M1), accounted for 4.96% (female) and 3.93% (male) of the bile samples (Figure 5C). Small amounts of [^3^H]BPC157 were detected in feces, accounting for 0.63% (female) and 2.26% (male) of the total fecal radioactivity. The tritium water content was 30.1% (female) and 29.3% (male), and the content of [^3^H]proline (M1) was higher, accounting for 20.7% (female) and 30.2% (male) of the total radioactivity (Figure 5D). The contents of other metabolites in feces were all lower than 0.06% of the administered amount, and it was impossible to perform structural identification because of the extremely low content. These results suggest that BPC157 was rapidly metabolized into low levels of a variety of small peptide fragments, finally resulting in a single amino acid represented by [^3^H]proline, which entered the normal amino acid metabolism and excretion pathway in the body.

#### 2.3.4 Tissue distribution of BPC157 in rats

**FIGURE 6:**
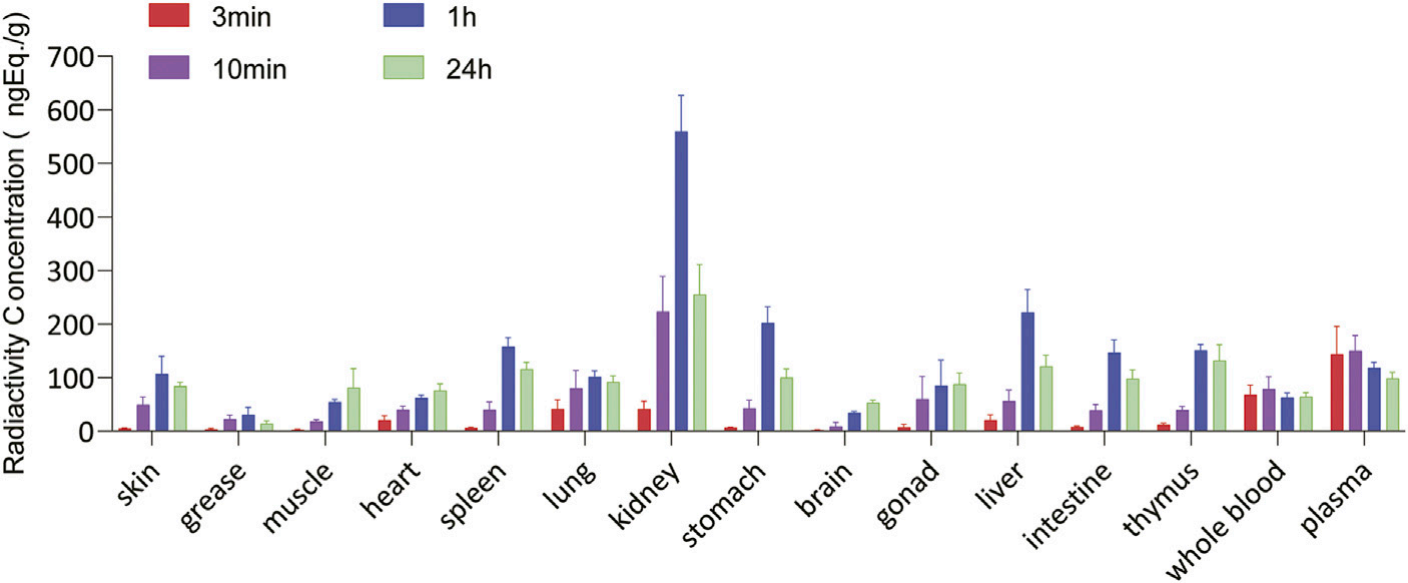
Tissue and organ distribution of [^3^H]BPC157 after a single intramuscular administration of 100 µg/300 μCi/kg of [^3^H]BPC157 in rats (mean ± SD *n* = 6 per time point), expressed as the content of BPC157 per gram of tissue/organ. Data are representative of three independent experiments.

The total radioactivity concentrations in the rat tissues were similar after a single IM injection of 100 µg/300 μCi/kg of [3H]BPC157. Tissue distributions at different time points are summarized in Figure 6. After 3 min of administration, total radioactivity concentration was detected in all rat tissues; however, it was significantly lower than that observed in the plasma. After 10 min of administration, the total radioactivity concentration increased significantly in all tissues, with the mean renal tissue concentration reaching 223 ng (Eq. µg/ml), which was higher than the mean plasma concentration (150 ng, Eq./ml). After 1 h of administration, the total radioactivity peaked in most tissues, and the average concentration in the kidney was the highest, reaching 560 ng (Eq. µg/ml), followed by that in the liver, stomach wall, spleen, and thymus. All average concentrations were higher than those in the plasma. The total radioactivity concentrations in the intestine, skin, and lungs were similar to those in the plasma, and the mean concentrations in the gonads, myocardium, skeletal muscle, brain, and body fat were all lower than the mean concentrations in the plasma. At 24 h after administration, the mean concentrations of total radioactivity in the kidney, thymus, liver, spleen, and gastric wall decreased significantly but were still higher than the mean concentrations in the plasma at the same time. The concentrations in other tissues were lower than the average concentration in the plasma and are presented in the descending order as follows: intestinal tract, lung, gonad, skin, skeletal muscle, cardiac muscle, whole blood, brain, and body fat. The total radioactivity concentrations in the kidney, liver, stomach wall, thymus, spleen, intestine, lung, skin, and body fat were reduced by approximately 50% compared with the peak concentration in the same tissue (1 h after administration).

## 3 Discussion

Pharmacokinetic evaluations are necessary and vital for the development of new drugs. To translate BPC157 into the clinic, we previously conducted preclinical safety studies and found that BPC157 was well tolerated and did not demonstrate serious toxicity (Xu et al., 2020). This study aimed to investigate the pharmacokinetics of BPC157. Experiments were performed to characterize the pharmacokinetics, absorption, distribution, metabolism, and excretion characteristics of BPC157 in rats and dogs.

We studied the pharmacokinetics of BPC157 after its IV and IM administration in rats and beagle dogs. According to the results, the elimination half-life (t_1/2_) of the prototype BPC157 was less than 30 min, and BPC157 showed linear pharmacokinetic characteristics in rats and beagles at all experimental doses. After IM injections of 20, 100, and 500 μg/kg of BPC157 in rats and 6, 30, and 150 μg/kg of BPC157 in beagles, plasma BPC157 reached its peak rapidly (within 9 min). The pharmacokinetic parameters of BPC157 did not significantly change after repeated administration of BPC157 compared to those observed after a single IM injection of the same dose administered daily for 7 days. The mean absolute bioavailability observed after IM injections was approximately 14%–19% in rats and 45%–51% in beagle dogs. In contrast to small-molecule compounds, peptide drugs demonstrate pharmacokinetic characteristics of short elimination half-life and poor metabolic stability *in vivo*. Generally, t_1/2_ values of peptide drugs range from a few minutes to an hour (Wang et al., 2016). The presence of a large number of proteolytic enzymes and peptidases in the body is the primary reasons for this phenomenon (Sharma et al., 2013). Therefore, in terms of the elimination half-life, BPC157 conformed to the characteristics of general peptide drugs. Our previous work has shown that IM injection of prototype BPC157 can effectively promote wound healing, and we aim to conduct clinical trials examining BPC157 for the treatment of severe trauma and burns in China. Nevertheless, extending the half-life of BPC157 and further improving its pharmacokinetic characteristics are important directions for the future development of this drug.

The radioisotope probe assay is a cost-effective and fast method for generating informative data for early preclinical/pharmacokinetic absorption, digestion, metabolism, and excretion studies of biotherapeutics (Roffey et al., 2007; Khalil et al., 2011; Chen et al., 2014). We labeled the proline of BPC157 with tritium and then studied the metabolism, excretion, and tissue distribution characteristics of BPC157 by examining the total radioactivity. The results of the excretion experiment showed that the main excretory pathways of BPC157 involve the liver and kidney, which was also consistent with the excretion characteristics of peptide drugs (Czock et al., 2012; Li et al., 2015). The tissue distribution results showed that the radioactivity intensity in most tissues peaked 1 h after administration, which was slightly later than the peak time of the total radioactivity concentration in plasma (0.167 h). The peak concentrations of radioactivity in the kidney, liver, stomach wall, thymus, and spleen were significantly higher than those in the plasma. The concentrations in the intestinal tract, lungs, and skin were similar to those in the plasma, followed by those in the gonads, cardiac muscle, skeletal muscle, and whole blood. The concentrations were lowest in the brain and body fat. These results suggested that BPC157 can enter tissues and cells to perform biological functions.

Determination of metabolites was the most challenging aspect of this study. The metabolism of peptides and proteins usually starts from the action of endopeptidase and then undergoes multi-step enzymatic degradation to produce the final metabolite amino acids, which enter the amino acid pool *in vivo* (Vugmeyster et al., 2012). In rat plasma, we identified six radioactive components, in addition to the prototype [^3^H]BPC157, and their structures were predicted by LC-MS/MS molecular weight identification and comparison with standards. Through the analysis of possible hydrolysis sites, we predicted the metabolic process of BPC157 and proved that BPC157 was finally metabolized into a single amino acid, represented by [^3^H]proline, in plasma, urine, and feces. These results show that BPC157 conforms to the metabolic process of peptide drugs, further proving its metabolic safety. However, analysis of the proportions of various metabolites in plasma over time once again suggested a short half-life and rapid degradation of prototype BPC157. In addition, we did not conduct metabolite analysis in tissues, especially in target organs, owing to the small sample size. The analysis of metabolites in tissues is important for further pharmacodynamic examination of BPC157 and explanation of its efficacy.

In conclusion, the present study is the first systematic report evaluating the pharmacokinetics, tissue distribution, metabolism, and excretion of BPC157. Many methodological validations were not included because of the limited space of the article. The results showed that the pharmacokinetic characteristics of BPC15 were consistent with the general properties of peptide drugs. In the future, we will conduct clinical trials for examining BPC157 for the treatment of severe trauma and burns. The observations of the present study and previous safety evaluation and pharmacodynamic research will provide basic information for further comprehensive clinical research. This study also provides a reference for the development of various peptide drugs.

## 4 Materials and methods

### 4.1 Test article and materials

BPC157 was synthesized and purified *via* HPLC in our laboratory with 99% purity. This compound was sterilized and lyophilized to meet the regulatory requirements of preclinical studies. [3H]BPC157 was synthesized by Moravek Biochemicals Inc. The specific radioactivity was 71.7 Ci/mmol, the radioactive purity was 99.6%, and the total amount was approximately 10 McUrie. The tritium labeling sites were located on two prolines.

BPC157 solution for administration was prepared by diluting the required amount of concentrated BPC157 solution in 0.9% NaCl injection solution prior to administration. The clinical dose of 200 µg/person/day of BPC157 was converted to 20 μg/kg for rats and 6 μg/kg for dogs. Based on its conversion according to body surface area and detection sensitivity, 100 µg/300 μCi/kg [3H]BPC157 was used for tritium labeling experiment in rats, 20, 100, and 500 μg/kg of BPC157 was used for unlabeled experiment in rats, and 6, 30, and 150 μg/kg of BPC157 was used for unlabeled experiment in dogs.

Warfarin sodium was purchased from Tokyo Chemical Industry Co. Ltd., Lot: 340JE. Heparin sodium and normal saline were obtained from Sinopharm Chemical Reagents Co. Ltd. (Shanghai, China). 3H scintillation solution was purchased from R.J. Harvey Co. (Tappan, NY). Ultima Gold scintillation solution was purchased from Perkin Elmer (Waltham, MA, United States). HPLC-grade acetonitrile, formic acid, and methanol were obtained from Merck (Darmstadt, Germany). HPLC-grade water was produced using a Milli-Q^®^ ultrapure water purification system (Bedford, MA, United States).

### 4.2 Animals

Approximately six-week-old SD rats weighing approximately 220 g were purchased from Beijing Vital River Laboratory Animal Technology Co., Ltd. The rats were maintained in an animal room with an air-conditioned barrier system at an ambient temperature of 25°C ± 2°C, relative humidity of 50% ± 10%, and a 12 h light/dark cycle. Ten-to-twelve-month-old beagle dogs weighing between 9.8 and 12.8 kg were purchased from YaDong Experimental Animal Research Centre, Nanjing, China. The dogs were raised in an open feeding farm under conditions involving natural light. The animals were provided with *ad libitum* access to clean drinking water and a standard pellet diet. The dogs were acclimatized to the housing conditions for at least 7 days prior to the initiation of the experiment. All animals were treated humanely, and all studies were carried out in accordance with good laboratory practice (GLP) (China Food and Drug Administration, CFDA) guidelines for nonclinical laboratory studies of drugs issued by the National Scientific and Technological Committee of the People’s Republic of China. Animal care and welfare were performed in accordance with the Guide for the Care and Use of Laboratory Animals.

### 4.3 Pharmacokinetic parameters in Sprague-Dawley rats after intravenous and intramuscular administration

A total of 324 SD rats were randomly divided into five groups, including 66 rats in group one, 60 rats each in groups two to four, and 78 rats in group five, with each group comprising half male and half female subjects. Group one was administered 20 μg/kg BPC157 saline solution intravenously. Groups two, three, and four were administered 20, 100, and 500 μg/kg BPC157 saline solutions *via* single IM injections, respectively. Group five was administered 100 μg/kg BPC157 normal saline solution by IM injection once a day for seven consecutive days. Blood samples were collected from rats in groups one to four at the corresponding time points before (0 h) and within 6 h after BPC157 administration. Blood samples were collected from rats in group five before the last three doses and within 6 h after the last dose. Three male and three female rats were selected at each time point, and approximately 7 ml of whole blood was collected by heart puncture. Blood was centrifuged at 4°C to obtain plasma and stored at 20°C until further analysis. The concentration of BPC157 in the animal plasma at different time points was determined by high-performance liquid chromatography-tandem mass spectrometry (LC-MS/MS). The calibration and quality control samples of BPC157 were prepared using animal plasma with K3EDTA as anticoagulant, and dextromethorphan was used as the internal standard of BPC157. The analyte and internal standard were extracted from 50 μl of plasma by solid phase extraction. BPC157 and internal standard were separated by reverse-phase chromatographic column, and the analyte was quantified by electrospray ionization (ESI) on a tandem four-stage mass spectrometer. The confirmed linear quantification range of BPC157 was 4.00 and 4,000 ng/ml. The pharmacokinetic parameters were calculated using the mean concentration and Watson LIMS software according to the non-atrioventricular model.

### 4.4 Pharmacokinetic parameters in beagle dogs after intravenous and intramuscular administration

In this part of the experiment, three male and three female beagles were examined for four cycles. In the first cycle, a normal saline solution (6 μg/kg) of BPC157 was administered intravenously. In the second and fourth cycles, the animals were administered 6, 30, and 150 μg/kg BPC157 saline solutions *via* single IM injections. In the third cycle, the dogs were administered 30 μg/kg BPC157 saline solution by IM injection once a day for seven consecutive days. Blood samples were collected at the corresponding time points before (0 h) and within 6 h of a single administration. Blood samples were collected from dogs administered multiple doses at corresponding time points before the first dosing (0 h), within 6 h after dosing, before the last three doses, and at corresponding time points after the last dosing. Approximately 3 ml of whole blood was collected at each time point through the venous plexus of the forelimb. The plasma was stored at −20°C for analysis.

### 4.5 Pharmacokinetic, tissue distribution, and excretion studies in rats administered radioactive-labeled BPC157

Thirty intact SD rats, six JVC rats, and six BDC rats (half male and half female subjects) were injected intramuscularly with 100 µg/300 μCi/kg of [3H]BPC157. Whole blood and plasma samples of six JVC rats were collected at 0.05, 0.167, 0.5, 1, 2, 4, 8, 24, 48, and 72 h after administration (three males and three females at each time point) for the examination of radio pharmacokinetics of total plasma. Six intact SD rats were used for urinary, fecal, and biliary excretion studies. Urine and fecal samples were collected from each rat at 0–8, 8–24, 24–48, and 48–72 h. Animal carcasses were collected 72 h after administration. Bile excretion was studied in six SD rats with BDC. Bile, urine, and feces were collected 0–72 h after administration. Tissue distribution of BPC was studied in 24 intact SD rats. The rats were euthanized, and tissue samples (brain, heart, kidneys, liver, spleen, lung, stomach, intestine, muscle, grease, ovaries, womb, testicles, and thymus) were collected at 3 min, 10 min, 1 h, and 24 h after administration (three males and three females at each time point). Male SD rats were administered a single IM injection of blank solvent (excipient), and biological samples, including whole blood, plasma, urine, feces, and tissues, were collected for background control. The radioactivity of the plasma, tissue, bile, urinary, and fecal samples was analyzed using a liquid scintillation counter.

### 4.6 Metabolism

Plasma, bile, urine, and fecal samples of intact SD rats or BDC rats after a single administration of [3H]BPC157 were analyzed by HPLC combined with a low-energy radionuclide detection technique to obtain the radiometabolite profiles of [3H]BPC157. The structures of the main metabolites of [3H]BPC157 in rat plasma, bile, urine, and feces were analyzed and identified using LC-MS/MS and standard molecular weight comparison.

### 4.7 Statistical analysis

All data are expressed as mean ± standard deviation. Pharmacokinetic parameters were evaluated using the WinNonlin software (version 5.3) according to a non-atrioventricular model. Linear regression was examined between AUC values obtained after BPC157 IM administration and BPC157 doses and between C_max_ values and BPC157 doses. The goodness of fit was estimated using the coefficients of determination (r^2^).

## Data Availability

The original contributions presented in the study are included in the article/Supplementary Material, further inquiries can be directed to the corresponding authors.
