# Supplementary figures and images for: Pharmacokinetics, distribution, metabolism, and excretion of body-protective compound 157, a potential drug for treating various wounds, in rats and dogs
Source: Front Pharmacol. 2022 Dec 14;13:1026182. doi: 10.3389/fphar.2022.1026182 (PMC9794587; doi:10.3389/fphar.2022.1026182)

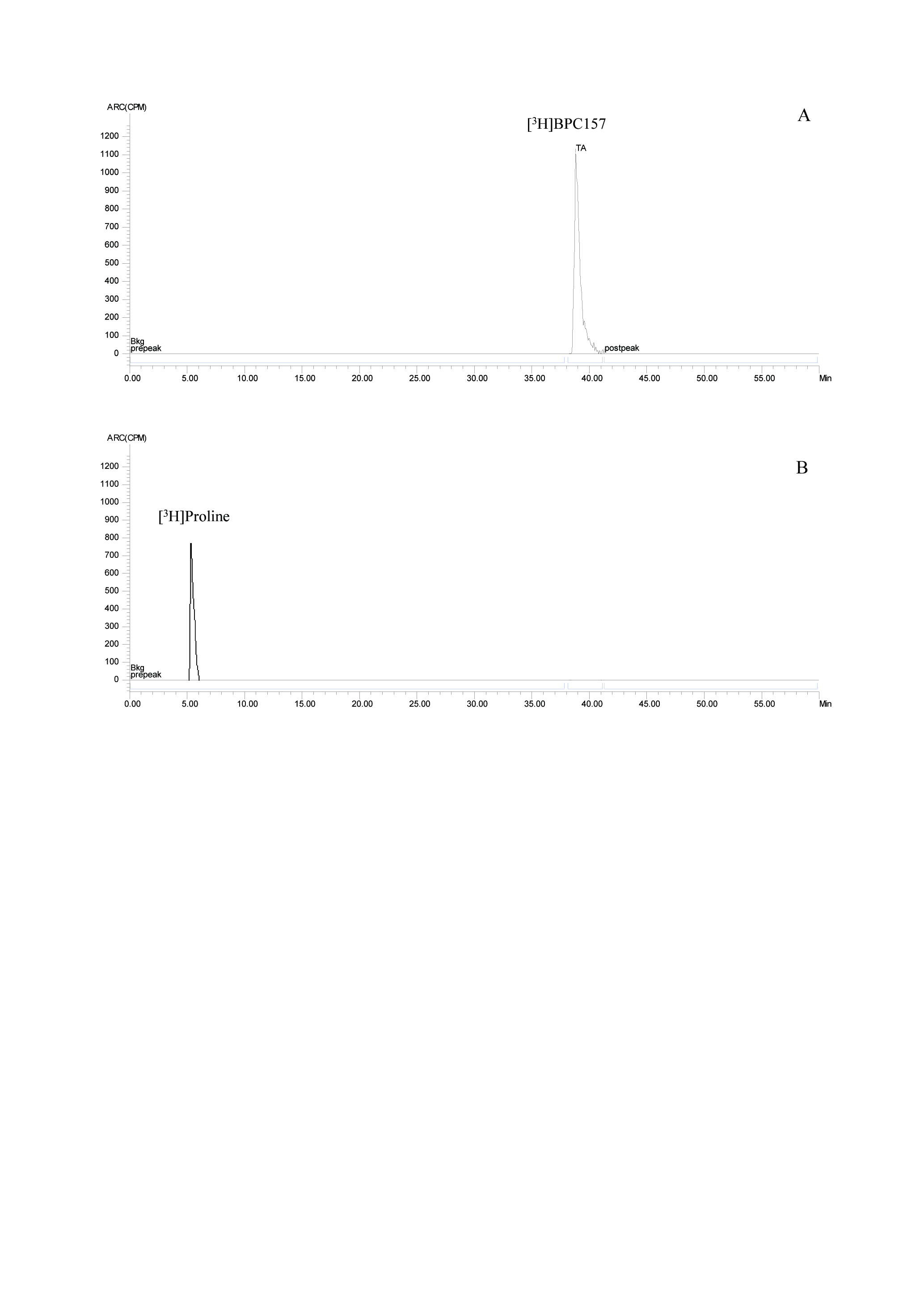

Supplement: Supplementary file 1 [file Image2.TIF]

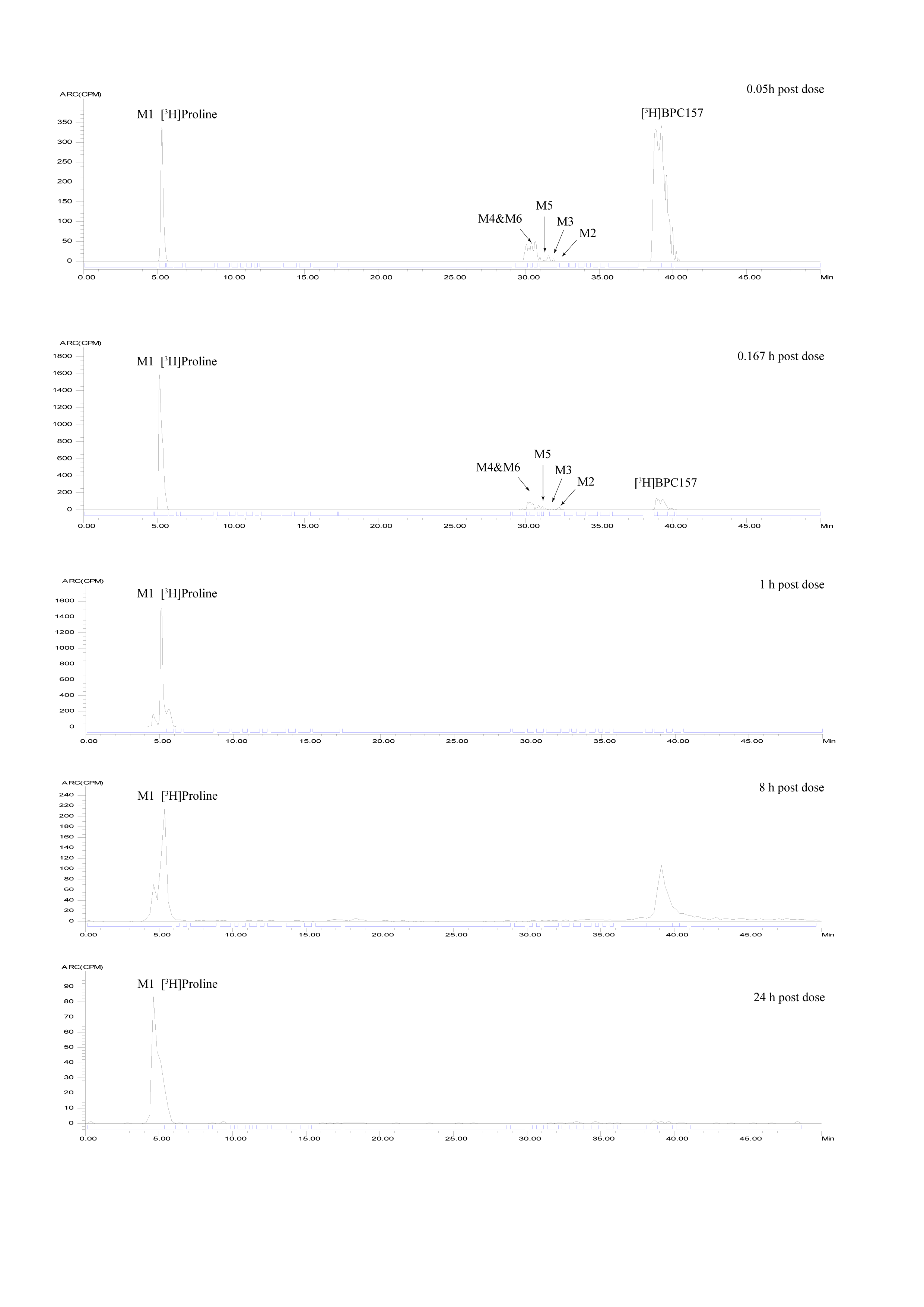

Supplement: Supplementary file 2 [file Image1.TIF]
